# Estimation of Ecosystem Services Value at a Basin Scale Based on Modified Equivalent Coefficient: A Case Study of the Yellow River Basin (Henan Section), China

**DOI:** 10.3390/ijerph192416648

**Published:** 2022-12-11

**Authors:** Haipeng Niu, Ran An, Dongyang Xiao, Mengmeng Liu, Xiaoming Zhao

**Affiliations:** 1 School of Surveying and Land Information Engineering, Henan Polytechnic University, Jiaozuo 454000, China; 2 Research Centre of Arable Land Protection and Urban-Rural High-Quality Development of Yellow River Basin, Henan Polytechnic University, Jiaozuo 454000, China; 3 School of Resources & Environment, Henan Polytechnic University, Jiaozuo 454000, China

**Keywords:** ecosystem services value, Yellow River Basin (Henan section), regional difference coefficient, social development stage coefficient, land utilization degree index, sensitivity coefficient

## Abstract

The value of ecosystem services is an extremely important parameter that reflects regional ecological benefits and resources. Estimating the value of ecosystem services is essential for regional land-use optimization, ecological construction, and biodiversity protection. In this study, Landsat-TM/ETM remote sensing images were used to analyze land-use data in 1990, 2000, 2010, and 2020 of the Yellow River Basin (Henan section), China, defined by natural boundaries. An equivalent factor method was used to construct a model to calculate the ecosystem services value that introduced grain yield, regional difference coefficient, and social development stage coefficient. Thus, land-use changes and evolution of ecosystem services values in the Yellow River Basin (Henan section) in the past 30 years were analyzed. Land use in the basin changed significantly from 1990 to 2020. Except for an increase in area of construction land, areas of other land-use types decreased. Cultivated land area first increased and then decreased, whereas the water area first decreased and then increased. The total value of ecosystem services in the study area fluctuated but increased overall by 43.82 × 10^8^ USD in the past 30 years. Spatially, the total value of ecosystem services was high in the southwest and low in the northeast. Among individual ecosystem service values, water conservation, gas regulation, and climate regulation accounted for a relatively high proportion of the total value. Regulation services were the main ecosystem service functions, followed by support and supply services, with cultural services accounting for the lowest proportion. Sensitivity coefficients of different land types in different periods were all less than one. Therefore, the value coefficients were reasonable, and the results were consistent with the actual situation of the study area. The study improves the method to estimate the ecosystem services value of the basin and also indicates ways to support ecological protection of the Yellow River Basin (Henan section), China.

## 1. Introduction

Ecosystem services refer to the ecological functions and products that can support the survival and development of human beings, which are produced by the ecosystem and the ecological process [1]. The services involve the structure, processes, and functions of ecosystems [2]. Ecosystem services values (ESV) are the benefits that humans obtain directly or indirectly from ecosystems and are used to monetize the value generated by natural ecosystems [3]. Ecosystem services include regulation, culture, support, and supply services [4], and ESVs are important in assessing the coordinated development of the regional economy and ecological environment [5]. According to the Millennium Ecosystem Assessment Report, more than two-thirds of ecosystem services have been degraded worldwide, and the decline in ecosystem services is likely to continue over the next 50 years [4]. As ecological problems such as global climate change, water pollution, reduction in biological species, and waste of resources become increasingly prominent [6], accurate estimation of ESVs is essential for regional ecological environmental governance and protection.

Measurement of ESV is currently a hot spot of research; many scholars have evaluated the ecosystem services [7,8,9]. In 1997, some scholars attracted widespread attention by measuring the value of global ecosystem services [10]. Subsequently, the value of urban ecosystem services was analyzed, and improvements were made to the ESV assessment model [11]. The change in ESV based on land-use change was then analyzed [12,13]. Some scholars used the Invest model to study the ecosystem service function of the Heihe River Basin [14]. In China, research on measuring ESV began relatively late. On the basis of the value evaluation model, Xie et al. (2003) established an equivalent factor table according to the ecosystems and socioeconomic development in China and constructed an ESV evaluation system suitable for China [15]. Improvements to the table followed [16,17]. Those developments attracted widespread attention in ecosystem services research. Some scholars examined how ecosystem services values were calculated and analyzed the connotation and development of ecosystem service functions [18]. Although much research has been conducted on methods to evaluate ESVs, a unified evaluation standard has not yet been formed [19]. Currently, ESVs are primarily measured by the functional value method [20,21,22] and the equivalent factor method [23,24,25]. According to the structure, functions and processes of different ecosystems, the functional value method converts the amount of material into a unified monetary value by using an evaluation model or multiple ecological equations. The method can objectively reflect ecological processes. However, owing to different service functions and evaluation indices of different ecosystems, results of evaluations are not intuitive, and data are difficult to obtain [26,27]. The equivalent factor method requires a relatively low amount of data and can be applied to the valuation of global, regional, and individual ecosystems [28]. However, the method provides a static assessment and does not consider spatial–temporal differences in ecosystem service functions, whereas ecosystem services change in time and space [29]. The equivalent factor method is based on a national average, and it is necessary to adjust the value coefficients for the ecosystems at the small scale. Different methods to improve the equivalent factor method have been proposed. The socioeconomic value created by per unit area of grain production was used to revise the assessment method and calculate the ESV of the Dongting Lake wetland [30]. The ability and willingness to pay have also been introduced to revise the value coefficients [31]; Liu et al. (2011) used changes in grassland biomass and coverage to introduce a spatial heterogeneity coefficient to assess the ESV of grassland [32]. Zhang (2012) corrected the value coefficient by using population density and ecological location to estimate farmland ESV [33]. Although many studies have revised equivalent value coefficients at different scales, most of the previous revisions do not comprehensively consider differences in population, economy, natural environment, biodiversity, spatial location, and other aspects. In addition, for different ecosystem types in different regions, methods to measure ESV are not the same.

Watersheds have a complex ecosystem structure and are closely associated with human production and activities. In watersheds, natural processes and human activities strongly interact [34]. The Yellow River is the second largest river in China, and the Yellow River Basin is important in the economic development and ecological security of China, particularly its ecological functions [35]. However, because of the long-term influence of traditional ideas and rapid population expansion in the basin, problems such as deterioration of the ecology and environment, serious deforestation, and annually increasing soil erosion have developed. Such problems are the main factors restricting sustainable economic development, and the basin has gradually evolved into an area with serious soil erosion in China [36,37]. In 2019, a major strategy of ecological protection and high-quality development in the Yellow River Basin was implemented, which had positive implications for the ecological quality of the Yellow River Basin. Currently, progress has been made on research on ESVs in the Yellow River Basin [38,39]. However, the provincial administrative boundary generally defined the research unit in previous studies, and research is lacking on the ESV of key regions in the Yellow River Basin with the natural boundaries defining the research unit. Moreover, because of the serious soil erosion in the middle reaches of the Yellow River Basin (Henan section), the downstream ecological flow is relatively low [40]. Therefore, in this paper, to explore changes in land use and ESVs in the Yellow River basin (Henan section), remote sensing and Geographic Information System (GIS) technology were used with the research unit defined by the natural boundaries as the research unit of the basin. Such an approach is essential to clarify the problems between land use and the ecology and environment, ecological protection, and sustainable development of the Yellow River Basin.

In this paper, the Yellow River Basin (Henan section) defined by natural boundaries was the research unit; land-use data in 1990, 2000, 2010, and 2020 were interpreted based on Landsat-TM/ETM remote sensing images, and the equivalent factor method was used to estimate the ESVs. However, because the equivalent factor method is based on the national average, the value coefficients needed to be adjusted for small-scale ecosystems. On the basis of the Costanza et al. (1997) evaluation model and with reference to the relevant studies of Xie et al. (2015), the coefficients of grain yield, social development stage, and regional difference were introduced to revise the value coefficient. Then, an evaluation model of ESV applicable for the Yellow River Basin (Henan section) was constructed, and the changes in ESV over 30 years (1990–2020) were explored. The goal of the study was to provide ideas for the coordinated development of the eco-environment and economy in the Yellow River Basin (Henan section).

## 2. Materials and Methods

### 2.1. Study Area

**Figure 1:**
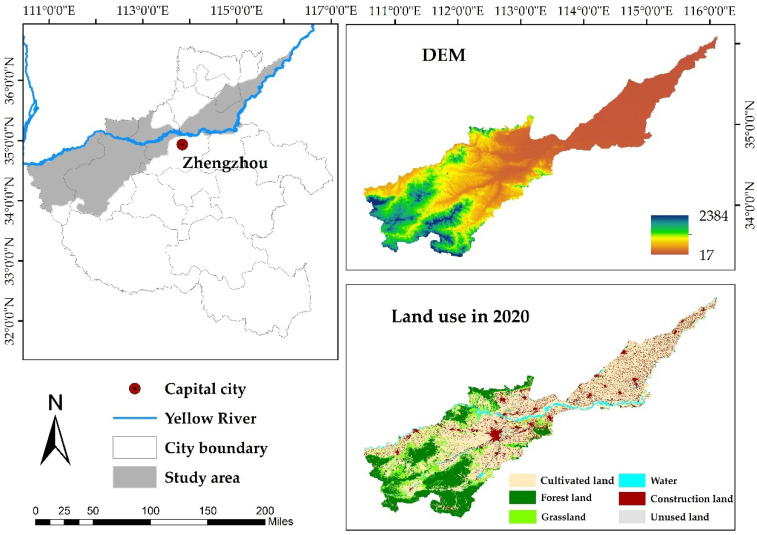
Schematic diagram of the study area. Note: The data of land-use cover in 2020 used in the figure were from Landsat 8 OLI remote sensing image data with a spatial resolution of 30 m. The DEM were from ASTER GDEM elevation data set with 30 m resolution.

The geographic coordinates of the Yellow River Basin (Henan section) are 33°N to 37N° and 110°E to 116°E. The Yellow River enters Henan Province from Tongguan, Shanxi Province, and from Lingbao City in the west to Taiqian County in the east. With a total length of 711 km, the basin covers 36,200 km^2^, accounting for 21.7% of the total area of Henan Province. The Yellow River Basin contains eight cities in Henan Province, including Sanmenxia, Xinxiang, Jiyuan, Luoyang, Jiaozuo, Kaifeng, Zhengzhou, and Puyang. The basin is mainly in the warm temperate zone, and most of the basin experiences a continental monsoon climate. According to 1 km monthly temperature and precipitation data published by the National Earth System Science Data Center (http://www.geodata.cn/ (accessed on 10 September 2021)), the annual average temperature in the basin is 5 °C to 15 °C, and the annual precipitation is between 600 and 1000 mm, with precipitation unevenly distributed in season and space. In general, the terrain is high in the west and low in the east, transitioning from mountains to low mountains and from hills to plains. The basin has dual attributes of the middle and lower reaches of the Yellow River. In 2019, the total Gross Domestic Product (GDP) of the study area was approximately 1772.80 × 10^8^ USD, and the per capita GDP was approximately 933.87 USD/person. The total population in 2019 was approximately 18.98 million; the average population density was approximately 519 people/km^2^, and the maximum population density was approximately 11,134 people/km^2^. The soil types are fluvo-aquic, loessial, Lou, cinnamon, and yellow-brown [41]. Location and characteristics of the Yellow River Basin (Henan section) are shown in Figure 1.

### 2.2. Data Collection and Processing

Data sources are shown in Table 1. Land-use data were from the Resource and Environmental Science and Data Center (https://www.resdc.cn/ (accessed on 17 October 2021)). Land-use cover data in 1990, 2000, and 2010 were primarily from Landsat-TM/ETM remote sensing images, and land-use cover data in 2020 were from Landsat 8 OLI remote sensing images. The overall accuracy of the dataset was 88.95%. Land use in the Yellow River Basin (Henan section) was divided into six types: cultivated land; forest land; grassland; water; construction land; and unused land. The basic geographic information data mainly included administrative boundaries and watershed boundaries, which were from the Resource and Environmental Science and Data Center. Meteorological data, including precipitation and temperature, were derived from monthly temperature and precipitation data published by the National Earth System Science Data Center (http://www.geodata.cn/ (accessed on 10 September 2021)) from 1990 to 2020, with a spatial resolution of 1 km. The digital elevation model data were from ASTER GDEM elevation data set with 30 m resolution published by Geospatial Data Cloud (http://www.gscloud.cn/search (accessed on 20 December 2021)). Socioeconomic development data were derived from relevant statistical yearbooks and the Resource and Environmental Science and Data Center. All statistical yearbooks were downloaded from CNKI (https://www.cnki.net/ (accessed on 5 April 2021)).

### 2.3. Research Methods

#### 2.3.1. Land Utilization Degree Index

The degree of land use cannot only reflect the attributes of land itself in land use but also can reflect the comprehensive influence of natural and human factors on land [42]. A comprehensive index of land-use degree can also effectively indicate changes in land use [43]. The grading and assigning system of land-use degree is shown in Table 2. The comprehensive index of land-use degree proposed by Zhuang and Liu [44] was calculated as follows:

(1) $$K=\sum_{i=1}^{n}A_{i}\times C_{i}\times 100$$

where K represents the comprehensive index of land-use degree, with K ∈ (100, 400); n represents the grading number of land-use degree; $A_{i}$ represents the grading index of the ith land-use type; and $C_{i}$ represents the area percentage of the ith land-use type in the study area.

#### 2.3.2. Estimation of Ecosystem Service Value in the Yellow River Basin (Henan Section)

The equivalent factor table is the basis to measure the value of ecosystem services. When the table is used directly, the estimated results deviate from the real situation of the study area because of obvious differences among different ecosystems [45]. Therefore, in this paper, the grain yield method, social development stage coefficient, and regional difference coefficient were used to correct the value coefficient.

(1) Revision of equivalent factor of ecosystem services value per unit area

In this study, ecosystem service functions were classified (Table 3) on the basis of the equivalent factor table of ESVs in 2015 [16]. However, because the table is based on the national average and does not define the value equivalent of construction land, it was necessary to revise those equivalent factors to the study area. Land-use types in the Yellow River Basin (Henan section) were compared with similar ecosystem types in the equivalent factor table. Among them, cultivated land corresponded to farmland; forest land corresponded to forest, and grassland corresponded to grassland, and the equivalent value of each land type was the average value of the second land type. Unused land corresponded to secondary land desert [46]. Water included canals, lakes, reservoirs, ponds, tidal flats, and shoals, and thus the average value of wetlands and waters in the secondary land types of the equivalent table was used [47]. There was not a corresponding ecosystem type of construction land, but its negative effects on the ecosystems could not be ignored. Therefore, the previous results were used to assign the equivalent value of construction land [48]. Thus, the equivalent factor table of ESV per unit area of the Yellow River Basin (Henan section) was obtained (Table 3).

(2) Calculation of economic value of grain yield per unit area

The value of the equivalent factor of the unit ESV is the economic value of the annual natural grain yield of the national average yield of 1 ha, which is equal to 1/7 of the market value of the national per-unit grain yield [15]. Among the eight cities in the Yellow River Basin (Henan section), Zhengzhou, Sanmenxia, Luoyang, and Xinxiang accounted for more than 75% of the basin area. Therefore, the planting area and output value of the four main crops of wheat, corn, rice, and soybean in the four cities were selected to revise the value coefficient. Moreover, because of the different prices of crops in different years, to avoid the effect of price changes on the calculations, the average value of long-term sequence grain prices was used as the constant price [46]. Therefore, the average value of grain prices of each crop in Henan Province from 2001 to 2020 was used in the following calculation:

(2) $$E_{t}=\frac{1}{7}\sum_{i=1}^{n}\frac{m_{i}p_{i}q_{i}}{M}\;\;\;(i=1,\cdots ,n)$$

where i represents the type of crop; $E_{t}$ represents the economic value of grain yield per unit area (USD/ha); $m_{i}$ represents the planting area (ha); $p_{i}$ represents the average price of grain (USD/t); $q_{i}$ represents grain yield per unit area (t/ha); and M represents the total planting area of crops (ha). The calculation results are shown in Table 4.

(3) Correction of social development stage coefficient of ecosystem services value in the Yellow River Basin (Henan section)

The coefficient of social development stage was introduced in this paper to modify the value coefficient of ecosystem services in the Yellow River Basin (Henan section). The Engel coefficient and Pierre growth curve model were selected to construct the coefficient of social development stage. The specific formulas were the following:

(3) $$P_{t}=\frac{l_{\mathrm{study}\;\mathrm{area}}}{l_{\mathrm{national}}}$$

(4) $$l_{\mathrm{study}\;\mathrm{area}}=l_{1}h_{1}+l_{2}h_{2}$$

(5) $$l_{\mathrm{national}}=l_{3}h_{3}+l_{4}h_{4}$$

(6) $$l=\frac{L}{1+ae^{-b\left(1/E_{n}-3\right)}}$$

where $P_{t}$ represents the coefficient of social development stage; $l_{1}$ and $l_{2}$, respectively, represent the coefficients of social development stages in urban and rural areas of the study area; $h_{1}$ and $h_{2}$, respectively, represent the proportions of urban and rural populations in the study area; $l_{3}$ and $l_{4}$, respectively, represent the coefficients of urban and rural social development stages; $h_{3}$ and $h_{4}$, respectively, represent the proportions of urban and rural populations in China; l is the growth characteristic parameter and represents the social development stage coefficient related to the willingness to pay; L is the maximum value of l and represents the willingness to pay in the stage of rich society and is equal to 1; $E_{n}$ is the Engel coefficient, including urban and rural areas; a and b are constants and are equal to 1; e represents the natural logarithm; and t represents the year. The results are shown in Table 5.

(4) Correction of regional difference coefficient of ecosystem service value in the Yellow River Basin (Henan section)

The coefficient of regional difference can reflect the differences caused by the combined effect of each factor in different regions [49]. Thus, the ESV coefficient was corrected from the perspective of regional differences. Biomass is the amount of material accumulated by plants per unit area [50], and the larger the biomass is, the stronger the ecosystem service function. In this study, net primary productivity (NPP) was used instead of biomass for the correction. Different models are used to calculate NPP, such as the Miami model [51], Chikugo model [52], and Thornthwaite Memorial model [51]. Among the models, the Thornthwaite Memorial model contains comprehensive environmental factors, and the correction factor adopts actual evapotranspiration, which can effectively reduce the error in calculations [53]. Therefore, the Thornthwaite Memorial model was used to calculate NPP according to the following formulas:

(7) $$NPP=3000\left[1-e^{-0.0009695\left(Z-20\right)}\right]$$

(8) $$Z=\frac{1.05R}{\sqrt{1+{\left(1+1.05R/H\right)}^{2}}}$$

(9) $$H=3000+25t+0.05t^{3}$$

where NPP represents the net primary production of natural vegetation (t∙ha∙a−1); e is the natural logarithm; Z represents the actual evapotranspiration (mm) of the study area in one year; R represents the annual precipitation in the study area; H represents the average evapotranspiration (mm) of the study area in one year; and t represents the average temperature (°C) of the study area in one year.

The ratio of natural vegetation net primary productivity of a certain type of vegetation to the average net primary productivity of all types of vegetation in the biological productivity index was used in the modification according to the following formula:

(10) $$Q_{t}=NPP/NPP_{mean}$$

where NPP and $NPP_{mean}$ represent the net primary productivity of natural vegetation and the average net primary productivity of all types of vegetation, respectively, and $Q_{t}$ represents the regional difference coefficient in the *t* year.

Equations (7)–(10) were used to obtain the regional difference coefficient of ESV in the Yellow River basin (Henan section) (Table 6).

(5) Construction of dynamic evaluation model of ecosystem services value

On the basis of ESV evaluation model [10] and according to the actual development of the Yellow River Basin (Henan section) combined with results in Xie et al. (2015), grain yield, social development stage coefficient, and regional difference coefficient were introduced to correct the value coefficient and construct the ESV evaluation model of the Yellow River Basin (Henan section). Combined with the annual land-use area of the study area (Table 7), the ESV of the Yellow River Basin (Henan section) was calculated. The model equations were the following:

(11) $$ESV=\sum_{i=1}^{n}\sum_{j=1}^{n}S_{i}\times E_{ij}\;\;\;(i=1,\cdots ,n;j=1,\cdots ,n)$$

(12) $$E_{ij}=e_{ij}\times E_{t}\times P_{t}\times Q_{t}\;\;\;(i=1,\cdots ,n;j=1,\cdots ,n;t=1,\cdots ,n)\;$$

where i represents the number of land-use types; j represents the number of ecosystem service function types; t represents the year; ESV represents the total value of ecosystem services in the Yellow River Basin (Henan section) (unit: USD); $S_{i}$ represents land-use area (ha); $E_{ij}$ represents the value coefficient of the study area (unit: USD/ha); $e_{ij}$ represents the equivalent factor value of the study area; $E_{t}$ represents the economic value of grain yield per unit area in the study area; $P_{t}$ represents the coefficient of social development stage in the study area; and $Q_{t}$ represents the regional difference coefficient of the study area.

#### 2.3.3. Sensitivity Coefficient of Ecosystem Service Value

The dependence of ESV on the value coefficient can be reflected by the coefficient of sensitivity (CS) [54]. The ESV coefficient per unit area of each land type was adjusted up and down by 50% to calculate the sensitivity coefficient. When CS > 1, the ESV of a land type is sensitive and elastic to the value coefficient, which indicates that the value coefficient is unreasonable, and the credibility of the assessment is low. When CS < 1, the land type ESV lacks elasticity in response to the value coefficient, which indicates that the value coefficient is reasonable, and the evaluation is highly credible. The formula to calculate the CS was the following:

(13) $$CS=\left|\frac{(ESV_{a}-ESV_{b})/ESV_{b}}{\left(E_{ia}-E_{ib}\right)/E_{ib}}\right|$$

where CS represents the coefficient of sensitivity; $ESV_{a}$ represents the adjusted ecosystem service value; $ESV_{b}$ represents the value of ecosystem services before adjustment; $E_{ia}$ represents the adjusted value coefficient; $E_{ib}$ represents the value coefficient before adjustment; and i represents the number of land-use types.

## 3. Results

### 3.1. Correction Results of Ecosystem Services Value Coefficient

**Figure 2:**
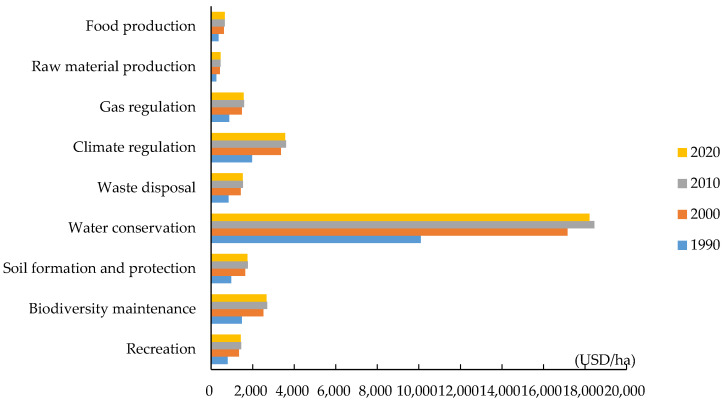
Changes of ESV coefficients in the Yellow River Basin (Henan section) from 1990 to 2020.

According to Equations (2)–(10) and results in Table 4, Table 5 and Table 6, the single service value coefficient ($E_{ij}$) of the ecosystem per unit area in the Yellow River Basin (Henan section) was calculated for gas regulation, climate regulation, waste treatment, water conservation, food production, raw material production, recreation, soil formation and protection, and biodiversity maintenance (Figure 2).

From 1990 to 2020, value coefficients of ecosystem services in the Yellow River Basin (Henan section) generally first increased and then decreased (Figure 2). In 1990, the social development stage in the basin was relatively low; the grain output was low, and the willingness to pay was insufficient, resulting in relatively low ecosystem service value coefficients. In 2000, with rapid development of the economy, the output value of grain per unit area increased, which increased the willingness to pay as the ability to pay gradually improved. Therefore, ecosystem value coefficients gradually increased. In 2010, with development of industrialization, the level of social development greatly improved, the grain output value further improved, and the willingness to pay increased further. Therefore, ecosystem service value coefficients continued to increase. In 2020, although the grain output value still increased compared with that in 2010, the willingness to pay decreased, resulting in a decline in value coefficients of ecosystem services.

### 3.2. Land Cover Change

**Figure 3:**
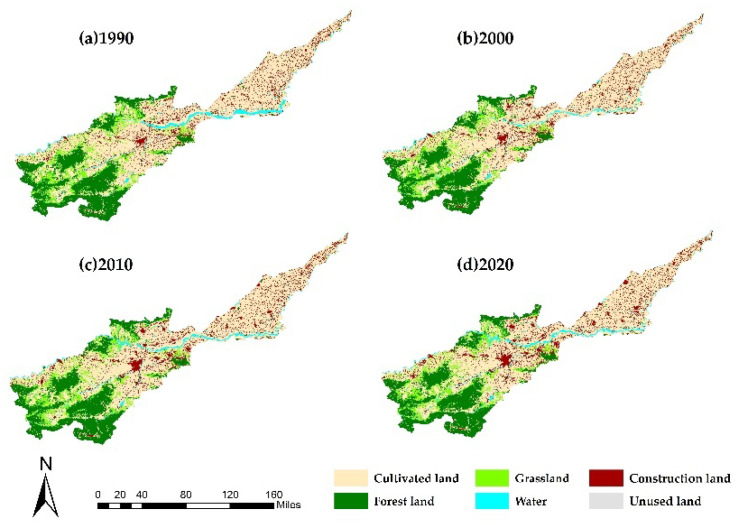
Land-use pattern in the Yellow River Basin (Henan section) from 1990 to 2020. Note: (**a**): Distribution of land-use pattern in 1990; (**b**): Distribution of land-use pattern in 2000; (**c**): Distribution of land-use pattern in 2010; (**d**): Distribution of land-use pattern in 2020. Land-use data were from the Resource and Environmental Science and Data Center with a spatial resolution of 30 m.

The spatial changes in land use in the Yellow River Basin (Henan section) in the four periods were visualized by ArcGIS 10.2 (from Esri Company in Redlands, California, USA) processing of land-use data (Figure 3).

**Figure 4:**
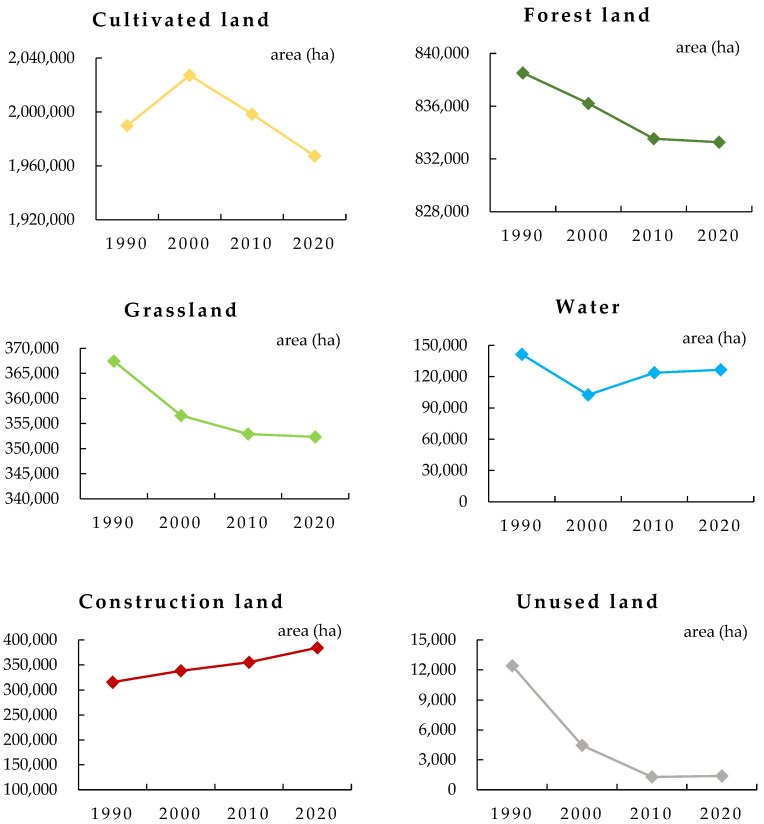
Change in area of different land-use types in the Yellow River Basin (Henan section) from 1990 to 2020 (unit: ha). Note: The solid points denote the land-use area, and different colors denote different land types.

The study area covered 3,665,061.54 ha, and the area of each land-use type was unevenly distributed with obvious differences (Table 7). Cultivated land was the largest land-use type, followed by forest land. The proportion of unused land was the smallest. Thus, the Yellow River Basin (Henan section) was dominated by cultivated and forest lands. In 1990, proportions of the two land types were 54.29% and 22.88%, respectively, accounting for 77.17% of the total area. In 2020, proportions of the two land types were 53.68% and 22.74%, respectively, accounting for 76.41% of the total area. From 1990 to 2020, except for an increase in construction land area, areas of other land-use types decreased (Figure 4). Areas of cultivated land and grassland decreased most substantially, by 22,588.74 ha and 15,101.11 ha, respectively. Area of cultivated land increased from 1990 to 2000 and then decreased continuously from 2000 to 2020. The main reason for the decrease is the conversion of cultivated land to forest land and construction land. Cultivated land to forest land is the implementation of the national policy of “returning farmland to forest,” and cultivated land to construction land is because of rapid urban expansion. Forest land area decreased continuously, decreasing by 5253.66 ha between 1990 and 2020. The decrease in forest land area is mainly due to the conversion of forest land to construction land and water area, but it can be seen from Figure 4 that the rate of forest land decrease is decreasing. Water area first decreased and then increased, but there was an overall reduction of 14,877.18 ha. Construction land area increased by 68,846.04 ha from 1990 to 2020, which was mainly due to the rapid expansion of urbanization. Such an increase is a common feature of all cities in China in the process of urbanization.

**Figure 5:**
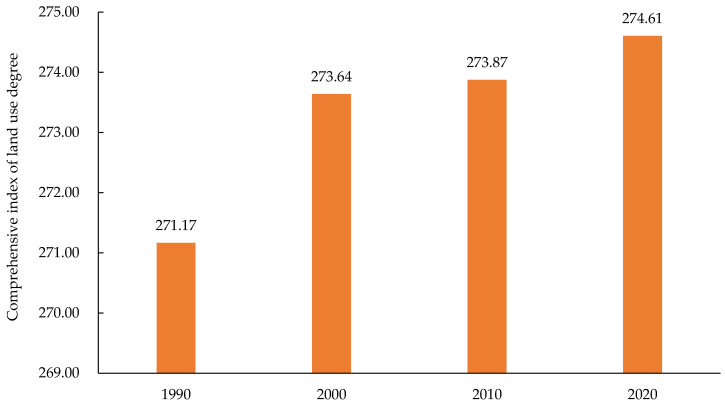
Comprehensive index of land-use degree in the Yellow River Basin (Henan section) from 1990 to 2020.

According to Equation (1), the comprehensive index of land-use degree was obtained (Figure 5). The comprehensive index of land-use degree of the Yellow River Basin (Henan section) in the four periods was between 271 and 275, and the index increased from period to period. From 1990 to 2000, the degree of land use changed greatly, increasing by 2.47. The land-use degree increased during this period because of population growth and rapid economic development. The change in the comprehensive index of land-use degree from 2000 to 2010 was small, only 0.23, and the change in land use was not obvious in the period, indicating that land use might have been in an adjustment period. The change in comprehensive index of land-use degree from 2010 to 2020 increased compared with that in the previous period, and the comprehensive level of land use in the study area improved.

**Figure 6:**
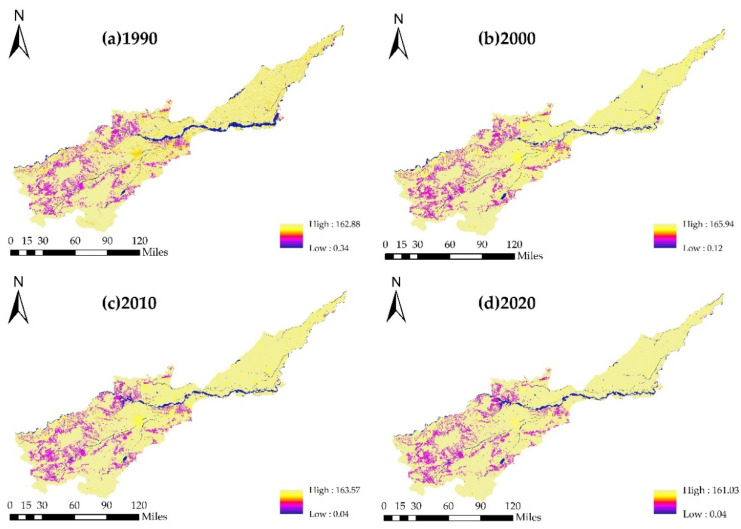
Spatial change of comprehensive index of land-use degree in the Yellow River Basin (Henan section) from 1990 to 2020. Note: (**a**): Distribution of comprehensive index of land-use degree in 1990; (**b**): Distribution of comprehensive index of land-use degree in 2000; (**c**): Distribution of comprehensive index of land-use degree in 2010; (**d**): Distribution of comprehensive index of land-use degree in 2020.

In general, the comprehensive index of land-use degree in the Yellow River Basin (Henan section) did not change much in space from 1990 to 2020 (Figure 6). The area with low land-use degree was concentrated in the middle reaches of the basin, which was where unused land, grassland, and water were concentrated. The area with high land-use degree index was in the lower reaches of the basin, where much of the land was cultivated. Because cultivated and forest lands were the dominant land-use types, the comprehensive index of land-use degree was higher in the area covered by those two land types than in areas of other land-use types.

### 3.3. Analysis of Changes in Ecosystem Services Value

#### 3.3.1. Changes in Total Ecosystem Services Values

With corrections of value coefficients, the evaluation model of ESV in the Yellow River Basin (Henan section) was obtained. According to Equations (11) and (12) of the model, total ESV of different land-use types in the study area from 1990 to 2020 were calculated (Table 8).

**Figure 7:**
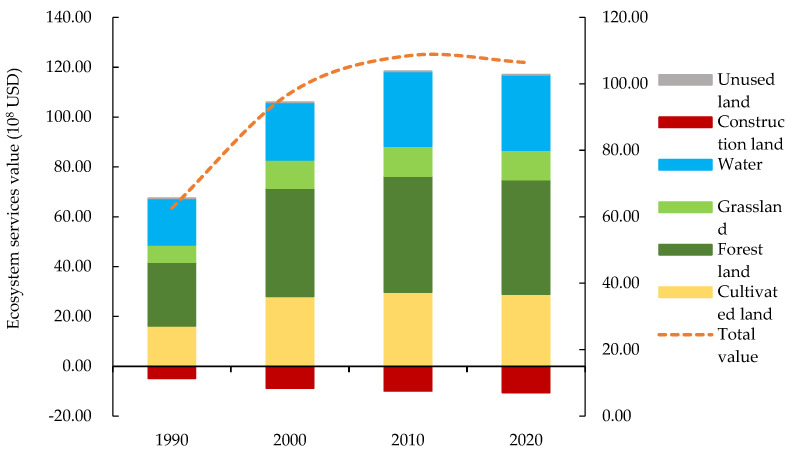
Ecosystem services value by land-use type in the Yellow River Basin (Henan section) from 1990 to 2020. Note: Different colors denote different land types. Broken lines denote total ESV changes.

**Figure 8:**
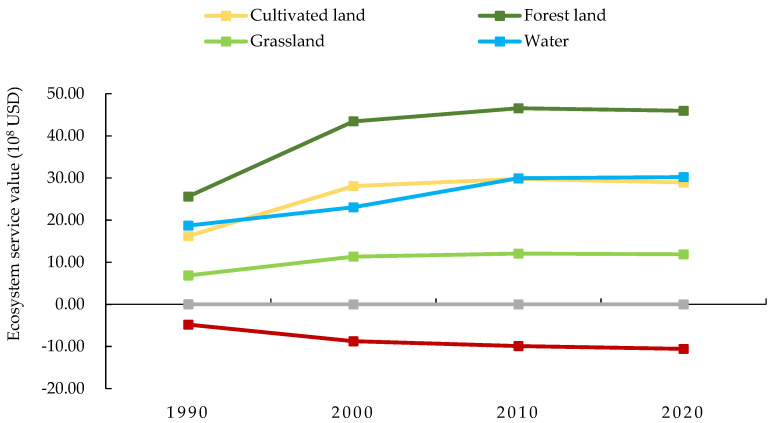
Changes of ESV by land-use type in the Yellow River Basin (Henan section) from 1990 to 2020. Note: The solid points denote ecosystem service value, and different colors denote different land types.

From 1990 to 2020, the value of ecosystem services in the Yellow River Basin (Henan section) fluctuated but generally increased, increasing from 62.61 × 10^8^ USD to 106.43 × 10^8^ USD, for an increase of 43.82 × 10^8^ USD or 69.98% (Table 8, Figure 7). The value of ecosystem services provided by unused land decreased by 0.02 × 10^8^ USD, a decrease of 79.81%, whereas the value of ecosystem services provided by other land types increased. The main contributors to the ESV in the basin were cultivated and forest lands, which accounted for 66.80% to 70.40% of the total value, indicating those lands were extremely important in ecosystem service functions of the basin. As shown in Figure 7 and Figure 8 and Table 8, except for construction and unused lands, the value of ecosystem services of other land types fluctuated but increased overall from 1990 to 2020. The ESV continued to increase from 1990 to 2010 but then began to decrease from 2010 to 2020, consistent with the trend in total ESV in the Yellow River Basin (Henan section).

Forest land provided the highest ESV. In the past 30 years, the ESV of forest land first increased first and then decreased. Overall, the ESV increased from 25.61 × 10^8^ USD in 1990 to 45.95 × 10^8^ USD in 2020, an increase of 79.41%. The value of ecosystem services provided by unused land accounted for the smallest proportion. The ESV decreased from 1990 to 2010 and then increased from 2010 to 2020. Construction land was the main reason for the loss in ESVs, and its ESV accounted for 7.67% to 9.93% of the total ESV. Construction land had a negative regulating effect on ecosystem services functions such as water conservation and waste disposal. In addition, the ESV loss in construction land increased, primarily because the area of construction land increased significantly from period to period. During the 30 years, construction land area increased by 68,846.04 ha, resulting in the loss of 5.76 × 10^8^ USD of ESV of construction land. The increase in area and loss in ESV were due to rapid development of an urban economy, and as the scale of construction land continued to increase, large amounts of cultivated and unused lands were converted.

**Figure 9:**
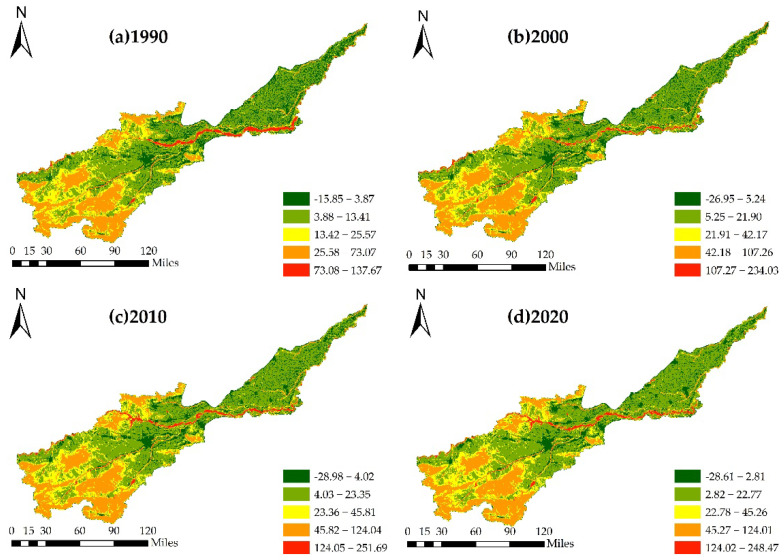
Spatial pattern of ESV in the Yellow River Basin (Henan section) from 1990 to 2020 (unit: 10^4^ USD). Note: (**a**): Distribution of ESV in 1990; (**b**): Distribution of ESV in 2000; (**c**): Distribution of ESV in 2010; (**d**): Distribution of ESV in 2020.

To highlight differences in the spatial distribution of ESV in the Yellow River Basin (Henan section), a 1 km × 1 km grid (based on the volume of data) with downscaling was constructed; the ESV of each grid was calculated. The natural breakpoint method was used to divide ESVs into five grades (Figure 9).

The spatial distribution of ESV was closely associated with land-use types (Figure 9). The value of ecosystem services was usually highest in areas where forest land and water were distributed. Because of the large area of cultivated land and the low proportions of unused and construction lands, the areas with low ESVs were mostly distributed in the northeast of the basin. From 1990 to 2020, owing to the rapid expansion of cities, the areas of with low ESVs increased significantly.

#### 3.3.2. Changes in Individual Ecosystem Services Values

The ESVs of soil formation and protection, waste treatment, gas regulation, climate regulation, food production, raw material production, water conservation, recreation, and biodiversity maintenance were calculated (Table 9). The ecosystem service functions of the Yellow River Basin (Henan section) were primarily reflected in water conservation, climate regulation, and gas regulation, which together accounted for more than 60% of the total value. The value of ecosystem services provided by food production, soil formation and protection, and biodiversity maintenance was the second most important. The ESV of raw material, waste disposal, and recreation accounted for a relatively small proportion of the total value, with all accounting for less than 5%. Values of the nine ecosystem service functions first increased and then decreased, which was consistent with the total value of ecosystem services in the study area.

**Figure 10:**
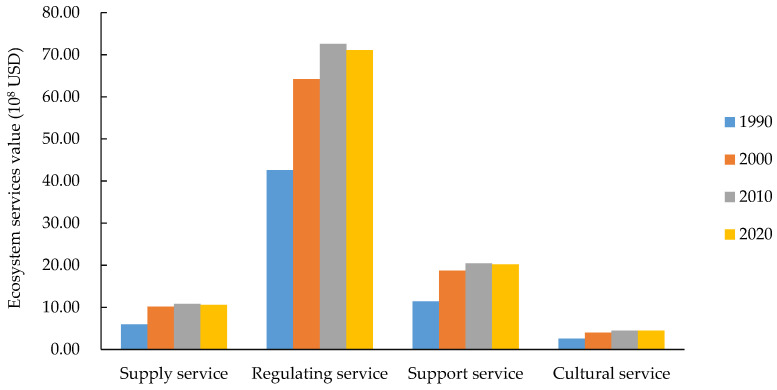
Ecosystem services values of the Yellow River Basin (Henan section) from 1990 to 2020 (By service types). Note: Different colors denote different years.

According to the first-level classification system of ecosystem services functions (Figure 10), the value provided by regulation service accounted for a large proportion, indicating that regulation service was the most important function in the Yellow River Basin (Henan section). The value increased by 28.51 × 10^8^ USD in the past 30 years, accounting for more than 60% in all years. The second most important was support service, accounting for 18.29% to 19.00%, whereas cultural service had the lowest value, accounting for only approximately 4% of the total value. The value of each service function was consistent with the total value, first increasing and then decreasing. The value of supply service, regulation service, support service, and cultural service increased from 5.97 × 10^8^, 42.60 × 10^8^, 11.45 × 10^8^, and 2.59 × 10^8^ USD to 10.62 × 10^8^, 71.12 × 10^8^, 20.22 × 10^8^, and 4.48 × 10^8^ USD, respectively.

### 3.4. Sensitivity Analysis

**Figure 11:**
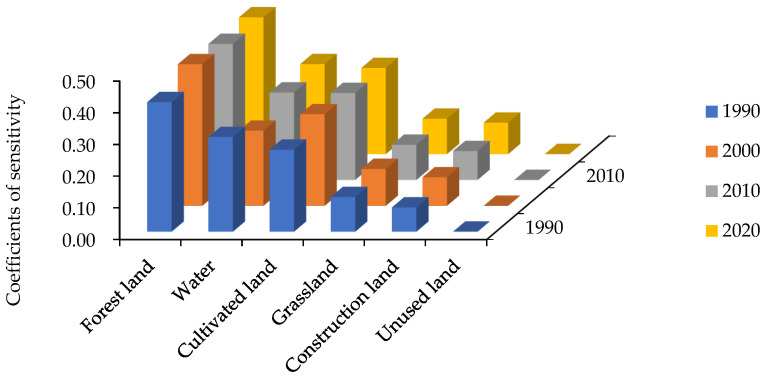
Coefficients of sensitivity of ESV in the Yellow River Basin (Henan section) from 1990 to 2020. Note: Different colors denote different years.

Equation (13) was used to calculate the coefficient of sensitivity of the ESV in the Yellow River Basin (Henan section) (Figure 11). After adjusting the value coefficient up and down by 50%, the CS values in different periods were all less than one, and the changes in CS in each period were not obvious. Therefore, the value coefficient of the study area was not flexible for ecosystem services functions. The CS value of forest land was the highest and was between 0.409055 and 0.447059, which indicated that for every 1% increase or decrease in the value coefficient of forest land, the value of ecosystem services provided by forest land increased or decreased by 0.409055% and 0.447059%, respectively. The CS value of unused land was the lowest at 0.000037, which indicated that when the value coefficient of unused land increased or decreased by 1%, the value of ecosystem services provided by unused land increased or decreased by 0.000037%. Therefore, the ESV of unused land had little effect on the total value. In addition, the CS values of other types of land use were all less than 0.3, indicating that changes in the value coefficients had a relatively small effect on the ESV of the Yellow River Basin (Henan section). Overall, the value coefficients calculated in this study were relatively reliable and consistent with the actual situation of the study area.

## 4. Discussion

Values of ecosystem services in cities along the Yellow River in Henan Province have been calculated. Chen (2020) adopted the equivalent factor method, corrected the regional coefficient, and obtained the value of ecosystem services per unit area [55]. Since this study obtained corrections for four periods when revising the economic value of grain per unit area, the value coefficients of ecosystem services have not been comprehensively evaluated. Therefore, the value coefficients of ecosystem services per unit area in the four periods were averaged and compared with the results of Chen (2020). The values of ecosystem services per unit area obtained after the coefficient corrections in this study were mostly higher than the results of Chen (2020). Although, the ESV per unit area of unused land was lower than 96.79%, the value coefficients per unit area of other land types were all higher. The value coefficient of ecosystem services per unit area of water area was 155.65% higher, whereas that of cultivated land was only 4.77% higher. Compared with the results of Chen (2020), the value coefficients of forest land, grassland, and construction land were all more than 70% higher. The reasons why the value coefficients in this study were higher might include the following: (1) Xie et al. (2003) proposed the original equivalence factor table suitable for China, which was later improved twice. In this paper, the equivalent factor table of Xie et al. (2015) was used, whereas Chen (2020) adopted the equivalent factor table before 2015. (2) In this study, the average value of the four periods value coefficient was compared with Chen’s value coefficient [55], and the average value is affected by the higher or lower value will result in different results. For example, the value coefficient per unit area in 1990 in this study was not significantly different, but the value coefficients increased each year since 2000. (3) Chen (2020) did not include the differences in social development and regional differences in coefficient correction, but these two factors play an important role in ESV assessment.

The price method based on unit service function and the equivalent factor method are the two methods primarily used to measure ESVs. In this paper, an improved equivalent factor method was used to calculate the ESV in the Yellow River Basin (Henan section) that modified the value coefficient of grain yield, the willingness to pay, and biomass factors. Compared with previous studies [56,57], in this paper, the differences in social development stages and location conditions are fully considered in the evaluation of ESV. In the estimation of the economic value of grain yield per unit area, the price of crops was expected to change over time, and thus the average price of crops in the past 20 years was used as an invariant price to eliminate the effect of price fluctuations on the evaluation. NPP is affected by many environmental factors [58], but temperature and precipitation are the main factors [59]. The commonly used models for calculating NPP are the Thornthwaite Memorial model [53], the Miami model [51] and the Chikugo model [60]. Lieth (1975) proposed the Miami model [51]. However, that model only considers the temperature and precipitation factors in the environmental factors, while NPP is also affected by other climatic factors, so it has obvious limitations [61]. Uchijima et al. (1985) proposed the Chikugo mode [60]. However, in the process of model derivation, NPP is calculated by evapotranspiration under the condition of sufficient soil moisture and very luxuriant vegetation, which makes many areas unable to meet this condition [62]. All the information does not include grassland and desert vegetation data [63]. Therefore, the model is not applicable to this study. Lieth et al. (1972) proposed the Thornthwaite Memorial model [53]. The evapotranspiration of the model is affected by a series of climatic factors such as temperature, air pressure, precipitation, solar radiation, and wind speed. The model contains more comprehensive environmental factors and adopts the actual evapotranspiration, so the net primary productivity is more reasonable [59]. Therefore, when correcting the regional difference coefficient, the Thornthwaite Memorial model is used to calculate NPP. When estimating NPP, the monthly precipitation and temperature data set was obtained from the National Earth System Science Data Center. Mask extraction technology was used to obtain the annual precipitation and annual average temperature of the Yellow River Basin (Henan section), which improves the accuracy of the data and thus that of the evaluation. This correction method is most suitable for the value assessment of small-scale, regional watershed ecosystems, and it greatly improves the reliability of assessment results and can better reflect changes in ecosystem services functions over time and space.

However, the corrected value coefficients in this paper are only preliminary because ESVs are also affected by comprehensive effects of biological productivity as landforms, vegetation, and soil [64]. In addition, the study was based on land-use remote sensing data, which are limited by image resolution, and errors cannot be avoided in the research process. However, the study was a long-term series of ESV analyses from a macro perspective, and the main purpose was to monetize the value of ecosystem services to reflect the ecological problems; those problems have limited effect on results.

The concept of ecosystem services as a means of linking social benefits to the basic functions of ecosystems has received extensive attention and is often incorporated into policymaking and legislation [65,66]. The study of ecosystem services in the Yellow River Basin has always been a hot issue, but the scale of most studies was at the city, county, township, or whole basin scale. Evaluation of ESV based on the natural boundaries of a certain section of the Yellow River Basin is relatively rare, even though the contributions of small-scale watershed ecosystems to ESVs is worth studying.

Because of the complex diversity and spatial heterogeneity of ecosystem services functions, there are many uncertain factors in measurements of ESV, which might have led to the low correlation between the economic value of grain yield per unit area and the total value of ecosystem services in the last period of this study. Therefore, in following research, the correction method will be further improved; in-depth research on other influencing factors will be conducted, and high-precision land-use remote sensing data will be identified in order to further improve the accuracy of assessment results and to identify the best measures to cope with changes in the ecology and environment.

## 5. Conclusions

Because of the current lack of research on small-scale watershed ecosystems, in this study, value coefficients were revised, accounting for regional differences and different levels of social development in the study area. Therefore, regional difference and social development stage coefficients were introduced to reveal the changes in regional ecosystem services. Then, changes in land use and ESV from 1990 to 2020 were analyzed. The major conclusions are summarized as follow:

(1) Cultivated and forest lands were the primary land-use types in the Yellow River Basin (Henan section). From 1990 to 2020, land use in the study area changed significantly, and areas of forest land, grassland, and unused land decreased. Construction land area increased. Water area first decreased and then increased, whereas cultivated land area first increased and then decreased. However, overall, cultivated land and water areas decreased. During the study period, the greatest change was in unused land, with the change rate reaching −88.82%. The area of forest land was the most stable over the 30 years, with a change rate of −0.63%.
(2) The total value of ecosystem services in the Yellow River Basin (Henan section) increased from 62.61 × 10^8^ USD in 1990 to 106.43 × 10^8^ USD in 2020, an increase of 43.82 × 10^8^ USD or 69.98%. The ESV fluctuated somewhat over time. The total value increased in the period 1990–2010 and began to decrease in the period 2010–2020. Spatially, the total value of ecosystem services was high in southwest areas and low in northeast areas. The land-use types in high-value areas were forest land and water, indicating that the contributions of forest land and water to the ESV were extremely important.
(3) Of the different ecosystem services functions, the contribution of regulating services accounted for a large proportion, and the ESV increased by 28.51 × 10^8^ USD in the past 30 years. Support service and supply service were also important. The lowest value was provided by cultural service, accounting for only approximately 4% of the total value. Among the individual ecosystem service values, water conservation, gas regulation, and climate regulation accounted for a relatively high proportion of the total value. During the study period, the pattern of change in values of each individual ecosystem service showed the same trend consistently increasing from 1990 to 2010 and then decreasing from 2010 to 2020.
(4) In analysis of the sensitivity index of the ESV, the smallest sensitivity coefficient was for unused land, indicating that changes in value coefficient of unused land had little effect on the total value of ecosystem services. The value coefficient of forest land ecosystems had the greatest effect on the total value of ecosystem services. In general, the CS values in different periods were all less than one, which indicated that the ecosystem services functions of the study were inflexible relative to the value coefficient. Therefore, the value coefficient was reliable, and the research results were credible.

## Figures and Tables

**Table 1:** Data sources.

Data Type	Source	Year
Crop sown area and yield	*Henan Statistical Yearbook*	1990–2021
Crop price	*National agricultural product cost benefit data compilation*	1990–2021
Proportion of population	*Henan Statistical Yearbook and China Statistical Yearbook*	1990–2021
Engel coefficient	*Henan Statistical Yearbook and China Statistical Yearbook*	1990–2021
Population density	Resource and Environment Science and Data Center	2019
GDP ^1^	Resource and Environment Science and Data Center	2019
Precipitation and temperature	National Earth System Science Data Center	1990–2020
DEM ^2^	Geospatial Data Cloud	2015
Land-use remote sensing data	Resource and Environment Science and Data Center	1990, 2000, 2010, 2020

^1^ Represents the gross domestic product; ^2^ represents the digital elevation model.

**Table 2:** Land-use grade index.

Land-Use Type	Cultivated Land	Forest, Grassland, Water	Construction Land	Unused Land
Grading index	3	2	4	1

**Table 3:** The equivalent factors of ESV per unit area in the Yellow River Basin (Henan section). Note: A negative value indicates the loss of ecosystem services value. The table is based on Xie et al. 2015, and the equivalent value of construction land comes from reference [48].

Ecosystem Service		Cultivated Land	Forest Land	Grassland	Water	Construction Land	Unused Land
Primary Classification	Secondary Classification						
Supply service	Food production	1.11	0.25	0.23	0.66	0.01	0.01
	Raw material production	0.25	0.58	0.34	0.37	0.00	0.03
Regulating service	Gas regulation	0.89	1.91	1.21	1.34	0.00	0.11
	Climate regulation	0.47	5.71	3.19	2.95	0.00	0.10
	Waste disposal	0.14	1.67	1.05	4.58	−2.46	0.31
	Water conservation	1.50	3.74	2.34	63.24	−7.51	0.21
Support service	Soil formation and protection	0.52	2.32	1.47	1.62	0.02	0.13
	Biodiversity maintenance	0.17	2.12	1.34	5.21	0.34	0.12
Cultural service	Recreation	0.08	0.93	0.59	3.31	0.01	0.05

**Table 4:** Economic value of grain yield per unit area in the Yellow River Basin (Henan section) from 1990 to 2020. Note: The data of planting area, unit yield of crop, and crop price were from relevant statistical yearbooks.

Year	Crops	Planting Area (10^4^ ha)	Unit Yield of Crop (t/ha)	Crop Price (USD/t)	Economic Value of Grain Yield per Unit Area (USD/ha)
1990	Wheat	83.20	3.51	261.33	143.14
	Rice	5.21	6.51	315.73	
	Corn	41.49	4.06	238.48	
	Soybean	8.77	1.20	527.89	
2000	Wheat	84.58	4.20	261.33	182.04
	Rice	5.61	6.85	315.73	
	Corn	44.60	5.05	238.48	
	Soybean	9.16	2.01	527.89	
2010	Wheat	84.30	5.29	261.33	212.27
	Rice	4.36	6.85	315.73	
	Corn	58.58	5.22	238.48	
	Soybean	7.92	2.20	527.89	
2020	Wheat	83.45	6.08	261.33	231.72
	Rice	1.47	6.62	315.73	
	Corn	67.78	5.64	238.48	
	Soybean	5.32	2.41	527.89	

**Table 5:** Coefficient of social development stage of ESV in the Yellow River Basin (Henan section) from 1990 to 2020. Note: The data of Engel coefficient and proportion of population were from relevant statistical yearbooks.

Region	Index	1990	2000	2010	2020
Study area	Urban Engel coefficient	0.55	0.35	0.31	0.25
	Rural Engel coefficient	0.55	0.40	0.33	0.25
	$h_{1}$	0.23	0.25	0.45	0.61
	$h_{2}$	0.77	0.75	0.56	0.40
	$l_{\mathrm{study}\;\mathrm{area}}$	0.23	0.40	0.53	0.74
National	Urban Engel coefficient	0.54	0.39	0.36	0.41
	Rural Engel coefficient	0.59	0.49	0.41	0.33
	$h_{3}$	0.26	0.36	0.50	0.64
	$h_{4}$	0.74	0.64	0.50	0.36
	$l_{national}$	0.22	0.32	0.41	0.57
	Social development stage coefficient	1.06	1.26	1.32	1.29

$h_{1}$ and $h_{2}$, respectively, represent the proportions of urban and rural populations in the study area; $h_{3}$ and $h_{4}$, respectively, represent the proportions of urban and rural populations in China; $l_{\mathrm{study}\;\mathrm{area}}$ and $l_{\mathrm{national}}$, respectively, represent the coefficients of social development stages in study area and China.

**Table 6:** Regional difference coefficients of ESV in the Yellow River Basin (Henan section) from 1990 to 2020. Note: The data of precipitation and temperature were from the National Earth System Science Data Center with a spatial resolution of 1 km.

Region	Index	1990	2000	2010	2020
Study area	Precipitation	668.10	728.20	669.08	694.80
	Temperature	13.21	13.46	13.62	13.88
	NPP ^1^	1019.44	1087.65	1021.11	1050.96
National	Precipitation	636.83	596.08	638.23	741.13
	Temperature	6.50	6.26	6.61	8.31
	NPP ^1^	975.90	926.85	977.64	1094.97
	Regional difference coefficient	1.04	1.17	1.04	0.96

^1^ Represents the net primary productivity.

**Table 7:** Area of land-use types in the Yellow River Basin (Henan section) from 1990 to 2020 (unit: ha).

Land-Use Type	1990	2000	2010	2020
**Cultivated land**	1,989,842.67	2,027,233.71	1,998,348.03	1,967,253.93
**Forest land**	838,524.6	836,208.81	833,532.75	833,270.94
**Grass land**	367,453.26	356,616.45	352,915.29	352,352.25
**Water**	141,417.09	102,458.25	123,748.2	126,539.91
**Construction land**	315,410.31	338,083.47	355,232.79	384,256.35
**Unused land**	12,413.61	4460.85	1284.48	1388.16

**Table 8:** Changes in ESV in the Yellow River Basin (Henan section) from 1990 to 2020 (unit: 10^8^ USD).

	Year	Cultivated Land	Forest Land	Grassland	Water	Construction Land	Unused Land	Total
ESV	1990	16.21	25.61	6.86	18.71	−4.80	0.02	62.61
	2000	28.09	43.43	11.33	23.05	−8.76	0.01	97.15
	2010	29.78	46.56	12.06	29.94	−9.90	0.00	108.44
	2020	28.94	45.95	11.88	30.22	−10.57	0.00	106.43
Change rate	1990–2000	0.73	0.70	0.65	0.23	0.82	−0.39	0.55
	2000–2010	0.06	0.07	0.06	0.30	0.13	−0.69	0.12
	2010–2020	−0.03	−0.01	−0.01	0.01	0.07	0.07	−0.02
	1990–2020	0.78	0.79	0.73	0.62	1.20	−0.80	0.70

The exchange rate of CNY/USD is calculated according to the average exchange rate in 2020 (i.e., 1 USD = 6.8996 CNY).

**Table 9:** Changes of ESV in the Yellow River Basin (Henan section) from 1990 to 2020 (unit:10^8^ USD).

Ecosystem Service		1990	Ratio (%)	2000	Ratio (%)	2010	Ratio (%)	2020	Ratio (%)
Supply service	Food production	4.13	6.59	7.06	7.26	7.53	6.95	7.34	6.90
	Raw material production	1.84	2.95	3.11	3.20	3.34	3.08	3.27	3.08
Regulating service	Gas regulation	6.37	10.17	10.72	11.04	11.51	10.62	11.29	10.61
	Climate regulation	11.62	18.55	19.36	19.93	20.89	19.26	20.59	19.35
	Waste disposal	3.08	4.92	4.57	4.71	5.04	4.65	4.79	4.50
	Water conservation	21.54	34.39	19.56	30.43	35.15	32.41	34.44	32.36
Support service	Soil formation and protection	5.97	9.53	9.97	10.26	10.75	9.91	10.57	9.93
	Biodiversity maintenance	5.49	8.76	8.76	9.02	9.72	8.96	9.64	9.06
Cultural service	Recreation	2.59	4.13	4.03	4.15	4.52	4.17	4.48	4.21
	Total	62.61	100.00	97.15	100.00	108.44	100.00	106.43	100.00

## Data Availability

The data presented in this study are available on request from the corresponding author.
